# Optogenetic spinal stimulation promotes new axonal growth and skilled forelimb recovery in rats with sub-chronic cervical spinal cord injury

**DOI:** 10.1088/1741-2552/acec13

**Published:** 2023-09-12

**Authors:** Sarah E Mondello, Lisa Young, Viet Dang, Amanda E Fischedick, Nicholas M Tolley, Tian Wang, Madison A Bravo, Dalton Lee, Belinda Tucker, Megan Knoernschild, Benjamin D Pedigo, Philip J Horner, Chet T Moritz

**Affiliations:** 1 Department of Rehabilitation Medicine, University of Washington, Seattle, WA 98195, United States of America; 2 Center for Neurotechnology, Seattle, WA 98195, United States of America; 3 Center for Neuroregeneration, Department of Neurological Surgery, Houston Methodist Research Institute, Houston, TX 77030, United States of America; 4 Department of Electrical and Computer Engineering, University of Washington, Seattle, WA 98195, United States of America; 5 Department of Physiology and Biophysics, University of Washington, Seattle, WA 98195, United States of America

**Keywords:** spinal cord injury, optogenetics, forelimb rehabilitation, axonal growth, angiogenesis

## Abstract

*Objective.* Spinal cord injury (SCI) leads to debilitating sensorimotor deficits that greatly limit quality of life. This work aims to develop a mechanistic understanding of how to best promote functional recovery following SCI. Electrical spinal stimulation is one promising approach that is effective in both animal models and humans with SCI. Optogenetic stimulation is an alternative method of stimulating the spinal cord that allows for cell-type-specific stimulation. The present work investigates the effects of preferentially stimulating neurons within the spinal cord and not glial cells, termed ‘neuron-specific’ optogenetic spinal stimulation. We examined forelimb recovery, axonal growth, and vasculature after optogenetic or sham stimulation in rats with cervical SCI. *Approach.* Adult female rats received a moderate cervical hemicontusion followed by the injection of a neuron-specific optogenetic viral vector ipsilateral and caudal to the lesion site. Animals then began rehabilitation on the skilled forelimb reaching task. At four weeks post-injury, rats received a micro-light emitting diode (µLED) implant to optogenetically stimulate the caudal spinal cord. Stimulation began at six weeks post-injury and occurred in conjunction with activities to promote use of the forelimbs. Following six weeks of stimulation, rats were perfused, and tissue stained for GAP-43, laminin, Nissl bodies and myelin. Location of viral transduction and transduced cell types were also assessed. *Main Results.* Our results demonstrate that neuron-specific optogenetic spinal stimulation significantly enhances recovery of skilled forelimb reaching. We also found significantly more GAP-43 and laminin labeling in the optogenetically stimulated groups indicating stimulation promotes axonal growth and angiogenesis. *Significance.* These findings indicate that optogenetic stimulation is a robust neuromodulator that could enable future therapies and investigations into the role of specific cell types, pathways, and neuronal populations in supporting recovery after SCI.

## 1. Introduction

Cervical spinal cord injury (SCI) often leads to significant sensorimotor deficits of the arms and hands resulting in the inability to perform daily tasks, and subsequent loss of autonomy and quality-of-life. Therapeutic electrical stimulation applied to the spinal cord via epidural (Lu *et al* 2016, Angeli *et al* 2018, Gill *et al* 2018, Wagner *et al* 2018, Darrow *et al* 2019) or transcutaneous electrodes (Gad *et al* 2018, Inanici *et al* 2018, 2021, Megía García *et al* 2020, Samejima *et al* 2022a) has been shown to promote impressive functional improvements in humans. Animal models in monkeys (Sharpe and Jackson 2014, Kato *et al* 2020, Greiner *et al* 2021, Barra *et al* 2022) and rats (Capogrosso *et al* 2016, Alam *et al* 2017) have shown similarly promising results that could help us understand the mechanisms behind improved functional recovery induced by electrical stimulation. Recently, it was found that epidural stimulation of the cervical spinal cord can improve upper limb function in individuals recovering from stroke, expanding the therapeutic applications of spinal stimulation (Powell *et al* 2023). Most likely, these beneficial results are due to enhanced activity within the spinal cord. Possible mechanisms that lead to long-term recovery lasting beyond the period of stimulation may be due to an increase in growth factors (Al-Majed *et al* 2000, 2004), neuroprotective factors (Shen *et al* 1999, Goldberg *et al* 2002, Yang *et al* 2014), and nutrient-rich blood flow that supports new axonal growth and lesion-bridging circuitry (Cox *et al* 1993, Harder *et al* 1998, Leybaert 2005, Mondello *et al* 2014).

While electrical stimulation activates all nearby neurons and glia (Tsui *et al* 2022), optogenetic spinal stimulation is an alternative method of stimulation that activates specifically neurons and not glia (Yizhar *et al* 2011). While the primary focus of this study is to investigate the effects of optogenetic spinal stimulation on recovery after SCI, our results also provide new insight into the individual roles of neurons, glial cells and pathways on restoring functional capabilities within the injured spinal cord. Understanding the individual effects of specific cell types and pathways will allow for more effective and efficient stimulation of various spinal cord-related deficits.

To date, long-term therapeutic optogenetic stimulation has not been applied to animal models of cervical SCI. This is largely due to the lack of an implantable device capable of providing optogenetic spinal stimulation for multiple weeks in awake, freely-moving rodents. Our group has designed an optogenetic spinal device capable of long-term stimulation and has reported on its safety and long-term stability (Mondello *et al* 2021). This device enabled us to conduct long-term optogenetic spinal stimulation in rats in the present study. Specifically, we investigated the effects of neuron-specific optogenetic spinal stimulation on forelimb recovery in rats with a sub-chronic, moderate hemicontusion. Neuron-specific stimulation is stimulation of only neurons, but not glia. This stimulation provides valuable insights into functional recovery due to stimulating a combination of excitatory and inhibitory neuronal cell types. These results provide a foundation for cell-type specific neuron stimulation in the future.

The current study documents the effects of optogenetic spinal stimulation caudal to the lesion site beginning six weeks post-injury and continuing for a total of six weeks. Stimulation was paired with rehabilitation on the forelimb reaching task (FRT) with the goal of guiding new circuitry towards accurate downstream targets that support enhanced FRT performance (Alaverdashvili *et al* 2008). Outcomes included the FRT, Irvine Beattie and Bresnahan task (IBB) (Irvine *et al* 2010, 2014), and limb-use asymmetry task (LUAT) (Schallert *et al* 2000, Gensel *et al* 2006). Optogenetic spinal stimulation led to significantly enhanced forelimb recovery on the FRT, but not the IBB or LUAT. Post-mortem tissue analysis indicated significantly more axonal growth in the spinal segments containing the lesion and caudal site of stimulation in the rats that received optogenetic stimulation compared to the unstimulated, sham-implanted control group. We also detected significantly more vasculature throughout the cervical spinal cord in the stimulated rats compared to control animals. Overall, these findings suggest that neuron-specific optogenetic stimulation is a powerful method for enhancing forelimb functional recovery after cervical SCI. The mechanisms supporting this recovery include axonal growth surrounding the site of stimulation, and angiogenesis throughout the cervical spinal cord. Our results indicate that optogenetic spinal stimulation should be further evaluated as a potential therapeutic for SCI.

## 2. Methods

**Figure 1.**
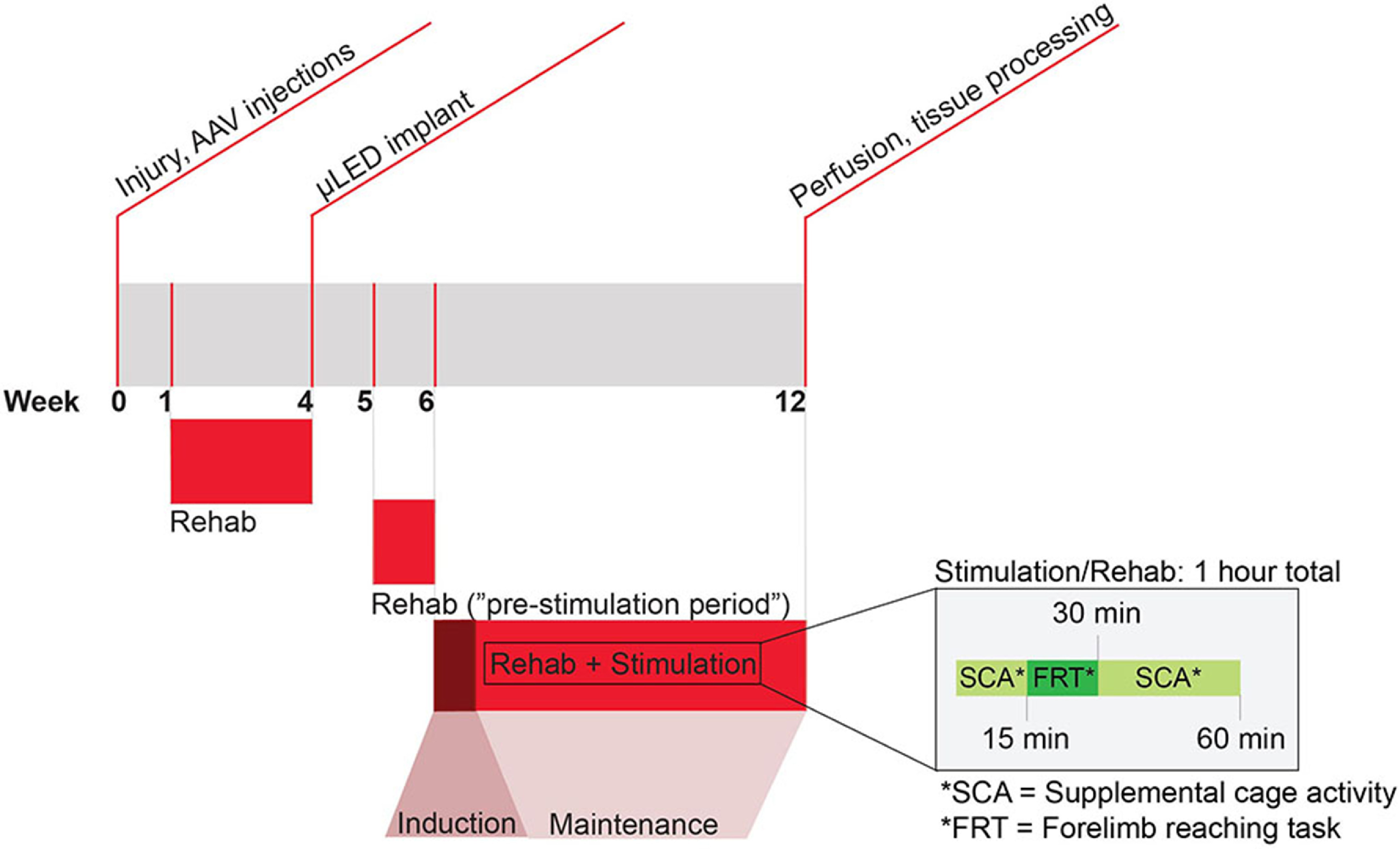
Experimental timeline and paradigm. Rats receive a hemicontusion at C4 and a spinal injection of *AAV2-hSyn-ChR2*(*H134*)*-YFP* at ipsilateral C6 during week 0. After a week of recovery, rats receive additional training on the forelimb reaching task for three weeks before a second surgery where they receive a *μ*LED implant over ipsilateral C6 (week 4). Two rats received an implant at two weeks post-injury. After a week of recovery, rats return to rehabilitative training during week 5. This week is considered the ‘pre-stimulation period’. During week 6–12 rats in the optogenetic stimulation group receive stimulation and rehabilitation. Week 6 is the ‘induction’ week, in which rats receive 1 h of stimulation daily for 4 d. The remaining 5 weeks of stimulation are ‘maintenance’ weeks in which rats receive stimulation for 1 h on only one day per week. Rehabilitation occurs in parallel with stimulation, depicted in detail within the inset. On week 12, rats were euthanized and spinal cord tissue was prepared for processing.

Overview: All animal procedures were approved by the University of Washington Institutional Animal Care and Use Committee. Eleven female, Long-Evans rats (250–350 g; Harlan, Indianapolis, IN) completed the long-term treatment experiments. Rats were divided across two groups: *optogenetic stimulation* (*N = 6*) and *unstimulated sham* (*N = 5*). Each rat began the study by acclimating to the three behavioral tasks after which they received a cervical hemicontusion (C4) and injection of an optogenetic virus ipsilateral and caudal to the lesion site (C6; figure 1). Most rats recovered for one week, followed by three weeks of rehabilitation before undergoing a second surgery four weeks post-injury. During the second surgery, animals received a micro-light emitting diode (*μ*LED) implant over C6 to deliver optogenetic light stimulation (Mondello *et al* 2021). After one week of recovery and one week of rehabilitation without stimulation, rats began receiving optogenetic stimulation at a total of six weeks post-injury. Stimulation was delivered for the subsequent six weeks. At the end of the study, rats were perfused and spinal cord tissue processed for multiple markers of interest.

Behavioral pre-training

Approximately two months prior to receiving a spinal injury, rats were trained to perform the skilled FRT (Alaverdashvili *et al* 2008) as well as acclimate to performing a forelimb use asymmetry task (Schallert *et al* 2000) and IBB cereal manipulation task (Irvine *et al* 2014). Prior to injury, all rats achieved at least two training days with a score of at least 70% success on the FRT in order to be included in the study. Once rats were proficient at completing all tasks, they were filmed to capture pre-injury baseline function.

SCI and intraspinal injections of ChR2 AAV vector

All rats received a C4 hemicontusion and intraspinal injections of the optogenetic vector *AAV2-hSyn-ChR2-YFP* (Deisseroth Laboratory, Chapel Hill, NC) at ipsilateral spinal segment C6 based on our prior work (Mondello *et al* 2021). The promoter utilized in this virus, human synapsin I, specifically targets neurons and not glial cells (Kügler *et al* 2003, Yaguchi *et al* 2013, Lee *et al* 2019). Rats were initially anesthetized with an intraperitoneal injection of ketamine (80 mg kg^−1^) and xylazine (10 mg kg^−1^). Supplemental dosages of ketamine were administered throughout the procedure as needed to maintain a surgical plane of anesthesia. Rats were placed on a liquid heating pad and shaved along the upper back and received an intradermal injection of lidocaine (1 mg kg^−1^) and bupivacaine (1 mg kg^−1^) at the site of incision, as well as a subcutaneous injection of enrofloxacin (5 mg kg^−1^). A subcutaneous injection of sterile saline (5 ml) was also provided to maintain hydration, and the eyes were coated with ophthalmic ointment to prevent dryness. The surgical field was cleaned with betadine and alcohol. An incision was made in the skin and through muscle layers covering vertebral segments C2–T2. A hemi-laminectomy was made over spinal segment C4 and a hemicontusion was produced using the Infinite Horizon Impactor (150 kdyne; Precision Systems and Instrumentation, LLC, Fairfax Station, VA).

Rats received AAV spinal injections immediately after the hemicontusion procedure. This process began with a partial laminectomy ipsilateral to the injury at the caudal aspect of spinal segment C6. Rats were positioned in a spinal stereotaxic frame (Kopf, Tujunga, CA). Two, 1.5 *μ*l deposits of *AAV2-hSyn-ChR2-YFP* (5.0 × 10^12^; UNC Vector Core) were then injected using a Hamilton syringe (Hamilton Company, Reno, NV) with a glass-pulled pipette tip attached to a model 5000 microinjection unit (AgnTho’s AB, Lidingö, Sweden). The targeted region of injection was just midline to the dorsal root entry zone and at a depth of 0.5 mm for both injection sites. A dwell time of 10 min was maintained for all injections.

Following the injections, gelfoam was placed over the exposed spinal cord at C4 and C6. The muscle and skin layers were sutured closed; the rats were then placed on a heating pad and provided intraperitoneal saline injections and a reapplication of ophthalmic ointment. Once awake, the rats received a subcutaneous injection of buprenorphine (0.05 mg kg^−1^) for pain management. This continued twice daily for the following two days. Weight and hydration were monitored daily for several days post-operatively.

*Forelimb rehabilitation—daily*

*Skilled FRT*

The skilled FRT is used to assess forepaw movement after SCI. Rats were placed in a clear arena with a slit leading to a platform 1 cm away from the arena containing a sugar pellet. Rats then (1) reached through the slit with their affected forelimb to (2) grab the pellet, (3) bring it into the arena, and (4) to their mouth to eat. The successful completion of this entire process is considered a ‘success’. If any of these steps could not be completed, animals ‘failed’ the attempt and could not eat the pellet. This task was performed 15 min per day, five days a week for the entirety of the study, acting both as rehabilitation and the primary outcome. Reaching success was scored during each of these rehabilitation sessions. The ‘handedness’ of each rat was determined prior to injury by identifying the favored forepaw and perfroming the injury to target the dominant limb.

Weekly videos of the FRT task were then used to determine the successful reaching attempts by researchers blinded to treatment groups. In cases where the performance on the filmed footage was a standard deviation above or below the weekly training average, the score from the day prior to filming was used. Video footage was also used to complete 12-point analysis (Alaverdashvili *et al* 2008), which breaks down forelimb reaches into 12 steps to analyze specific aspects of forelimb function. These include: (1) ‘Orient’—the rat’s ability to appropriately position themselves in front of the pellet, (2) ‘Limb-lift’—the rat’s ability to lift their forelimb with the palm surface in the vertical plane, (3) ‘Digits closed’—evaluation of whether the digits are flexed during limb lift, (4) ‘Aim’—the rat’s ability to aim their forepaw towards the pellet, (5) ‘Advance’—the rat’s ability to advance their forelimb towards the pellet, (6) ‘Digits Extend’—the rat’s ability to extend their digits toward the pellet at the end of the ‘advance’. (7) ‘Pronate’—the positioning of the paw over the pellet with their palm facing downward with digits extended, (8) ‘Grasp’—the rat’s ability to flex and close their digits around the pellet, (9) ‘Supinate I’—the rat’s ability to rotate their paw around the wrist to move into a vertical orientation, (10) ‘Supinate II’—continued rotation of the paw towards their mouth, (11) ‘Release’—the rat’s ability to open their digits and release the pellet into their mouth, and (12) ‘Replacing the limb’—the rat’s ability to return their forelimb to the floor.

Five weeks post-injury was considered the ‘pre-stimulation’ data point, as this occurred one week after the second surgery but just prior to stimulation. Therefore, this time point was used as the baseline for determining changes in forelimb recovery due to optogenetic or sham stimulation. During periods of stimulation beginning six weeks post-injury, all rats were connected to a stimulation cable and, if in the optogenetic stimulation group, received optogenetic stimulation during their forelimb reaching rehabilitation with the goal of helping to promote functional recovery. All rats required an average FRT score greater than 0% by six weeks post-injury to be included in the remainder of the study.

*Supplemental cage activity for extended forelimb use*

Rats were provided with a variety of rat-safe toys in their cage for 45 min, 5 days per week for the entirety of the study. This time, combined with the 15 min training on the FRT, amounts to approximately 1 h of forelimb activity each weekday. For the treatment group, this forelimb activity was paired with stimulation beginning at six weeks post-injury with the goal of promoting functional circuitry formation and limiting the potential for aberrant plasticity (figure 1).

*Forelimb rehabilitation—weekly*

*IBB cereal manipulation task*

This task was used to measure changes to proximal forelimb and fine digit manipulation once a week throughout the study (Irvine *et al* 2010, 2014). Animals were placed in a clear cylinder and provided both donut shaped cereal (Froot Loops, Kellogg’s, Battle Creek, MI) and spherical shaped cereal (Reese’s Puffs, General Mills, Minneapolis, MN). Rats were filmed manipulating each piece of cereal for consumption (three pieces of each type). An individual blinded to the treatment groups watched the video footage at 40% normal speed and analyzed the functional features of the effected forelimb using the published nine point scale (Irvine *et al* 2014). These features include: (1) ‘Predominant elbow position’—the rat’s level of elbow flexion/extension while eating: extended, partially flexed, or fully flexed, (2) ‘Proximal forelimb movements’—the amount of shoulder/elbow movement while eating: none, slight, or extensive, (3) ‘Contact non-volar support’—the rat’s use of non-volar surfaces to help support the cereal while eating: none, some, or almost always, (4) ‘Predominant forepaw position’—the rat’s common positioning of the ipsilateral forelimb while eating: clubbed, fixed and flexed, extended non-adaptable or partially flexed adaptable, (5) ‘Contact volar support’—the rat’s use of their ipsilateral forepaw’s volar surface to support cereal while eating: none, some, or almost always, (6) ‘Wrist movement during manipulation’—assessment of wrist movement while eating: yes or no, (7) ‘Cereal Adjustments’—the evaluation of the rat’s ability to produce successful, synchronized manipulations of both the ipsilateral and contralateral forepaw while eating cereal: none, exaggerated, subtle, (8) ‘Presence of digit movements’—assessment of each digit’s movement while eating cereal: non-contact, or contact manipulatory, scored as yes or no for each, (9) ‘Grasping method’—evaluation of the rat’s most common grasping technique (>50% of the time) for eating cereal: abnormal, sometimes normal, or almost always normal. The scores for each week and cereal type were averaged across all animals within each group.

*Forelimb use asymmetry task*

Animals were tested on a forelimb asymmetry use task once a week (Schallert *et al* 2000, Gensel *et al* 2006) to identify the frequency at which they used their affected versus intact forelimb to explore an arena. This is considered a gross assessment of proximal forelimb function. Rats were placed in a clear, acrylic cylinder and were observed for up to 5 min while a scorekeeper recorded the number of times rats reared up and made contact with the sides of the cylinder with either ipsilateral-only, contralateral-only, or both forelimbs (dual-limb) for a total of 20 contacts.

*μLED implant procedure*

Two to four weeks after receiving a spinal injury and viral injections, rats underwent a second surgical procedure to implant a *μ*LED as previously described (Mondello *et al* 2021). Two rats received their implant at two weeks post-injury instead of four weeks post-injury but received the same amount of rehabilitation as all other animals with no apparent effects related to this difference in timing. This variation was tested statistically by comparing recovery on the FRT between rats that were implanted at 2 versus 4 weeks post-injury at 7, 9, and 11 weeks post-injury (Mann–Whitney U-test; *p* = 0.248, 0.564, and 0.767, respectively).

To begin the procedure, rats were anesthetized with a ketamine (80 mg kg^−1^)/xylazine (10 mg kg^−1^) mixture. Supplemental dosages of ketamine were provided throughout the procedure to maintain a surgical plane of anesthesia. The upper back and top of head were shaved and cleaned with betadine and alcohol. Intradermal injections of lidocaine (1 mg kg^−1^) and bupivacaine (1 mg kg^−1^) were applied near the incision site and a subcutaneous injection of sterile saline (5 ml) was made for hydration. Ophthalmic ointment was applied to the eyes to prevent dryness and the rat was positioned onto a liquid heating pad. An incision was made over C2–T2 through the skin and muscle layers. A small amount of vertebral lamina was removed over caudal C6, the site of stimulation. A small hole was then drilled into ipsilesional and contralesional C5 using a Dremel tool with a .0210′′/.533 mm drill bit (Gyros Precision Tools). A 5/32 inch #000 flat fillister head screw (JI Morris) was inserted into the contralesional hole at C5 and a second screw passed through the hole of the *μ*LED implant and into the bone.

The LED portion of the implant was then positioned over the exposed spinal cord at ipsilesional C6 where the hemi-laminectomy and *AAV-ChR2* injection were performed in the prior surgery. The *μ*LED screw was affixed into the hole at ipsilesional C5 to anchor the implant into place. Kwik-Sil (WPI, Sarasota, FL) was applied to fill any space between the implant and top of the exposed spinal cord to minimize scar tissue accumulation. Next, UV-curable Fusio^TM^ liquid dentin (Pentron, Orange, CA) was applied over the top of the implant and contralesional lamina and then cured using an LED cure lamp (NSKI dental, Hoffman Estates, IL).

The catheter connecting the *μ*LED to the head-mounted pedestal was routed under the skin. The pedestal was then affixed to the top of the skull using a combination of microscrews (5/32 inch #000 flat fillister head screw; JI Morris), C & B Metabond Quick Adhesive System (Parkell, Edgewood, NY), and Ortho Jet Liquid Acrylic and Jet Denture Repair Powder (Lang, Wheeling, IL). The muscle layers and skin were sutured closed. Ophthalmic ointment was reapplied, and buprenorphine was administered subcutaneously twice daily for the following two days. Enrofloxacin (25 mg kg^−1^) was added to water bottles for 7 d to provide additional protection against infections.

*Therapeutic optogenetic stimulation*

Rats in the optogenetic stimulation group received stimulation beginning six weeks post-injury. The stimulation parameters were based on those determined to be thermally-safe in our previous work (Mondello *et al* 2021): 4 Hz pulse frequency, 5 ms pulse width, trains of stimulation delivered for 5 s on/15 s off for 1 h. The light intensity was set at movement threshold, as determined prior to each stimulation session. The first week of stimulation was considered the ‘induction’ week whereby rats received stimulation for four consecutive days. Stimulation weeks 2–6 were ‘maintenance’ weeks where stimulation was administered only on the first day of each week. During stimulation, rats received 15 min of supplemental cage activity, followed by 15 min of rehabilitation on the FRT. They then concluded with 30 min of supplemental cage activity (figure 1). Due to premature implant failures, all rats received stimulation up to at least 9 weeks post-injury with one rat continuing stimulation for one more session and two rats receiving two more sessions of stimulation up to 11 weeks post-injury. There were no detectable differences in recovery between the group of animals that received 4 weeks versus > 4 weeks of stimulation (Mann–Whitney U-test, *p* = 1.00). Any sign of infection occurring below the implant for rats in either group was cause for exclusion from the study.

*Histology*

*Perfusion and tissue sectioning:* After the 6th week of stimulation, rats received an intraperitoneal injection of Beuthanasia (200 mg kg^−1^) and were transcardially perfused using 0.9% sodium chloride, followed by 10% formalin. The cervical spinal cord was dissected and placed in 10% formalin overnight for 24 h at 4 °C. Spinal cord tissue was cryopreserved in 30% sucrose for 24 h at 4 °C. Just prior to freezing, the cervical spinal cord was divided into three blocks: ‘rostral’ (4 mm; above the lesion), ‘lesion’ (the lesion site including 1 mm of spared tissue above and below), and ‘caudal’ (4 mm; below the lesion; site of stimulation). Each block of tissue was embedded in O.C.T mounting compound (Sakura Finetek, Torrance, CA) and then placed in a mixture of dry ice and acetone to rapidly freeze. Tissue was sectioned into 40 *μ*m-thick cross sections using a cryostat.

*Assessment of AAV vector transduction*

To assess the location of viral transduction in the stimulated rats, tissue sections from cervical segment C6 were mounted onto Superfrost plus slides (Fisher Scientific, Hampton, NH) and covered with a coverslip using polyvinyl alcohol (PVA) mounting media (Sigma-Aldrich, St. Louis, MO). Sections were then imaged on a Zeiss Axio Zoom microscope (Zeiss, Jena, Germany) to confirm the presence of fluorescence of the reporter gene, YFP, indicating AAV transduction.

*Quantification of spared tissue after injury*

Sections from lesion site at C4 were mounted onto subbed Superfrost plus slides (Fisher Scientific, Hampton, NH) and stained with cresyl violet and myelin dyes as described in our previous work (Mondello *et al* 2015). Tissue was first fume-fixed onto slides for 24 h in a chamber containing 10% formalin. Tissue was then incubated for 5 min in water, followed by 70%, 95%, 100% ethanol (EtOH), and xylene. This process was subsequently repeated in the opposite order. Sections were then incubated for 10 min in myelin dye consisting of eriochrome cyanine R (Fluka, St. Louis, MO) and then differentiated in ammonium hydroxide and water. Next, sections were incubated in cresyl violet dye consisting of cresyl violet with acetate for 3 min (Sigma-Aldrich, St. Louis, MO). Sections were dipped ten times in 70% EtOH, and 95% EtOH then differentiated with glacial acetic acid and 95% EtOH. Sections were then incubated for 10 min in 100% EtOH followed by xylene, and coverslipped with Eukitt (Sigma-Aldrich).

FIJI (Schindelin *et al* 2012) was utilized to quantify the amount of spared tissue at the lesion site by identifying the lesion epicenter and determining the area of spared tissue within the ipsilesional hemicord excluding the lesioned tissue and dividing it from the total area of the ipsilateral hemicord (including the lesion; Samejima *et al* 2022b). To quantify spared rubrospinal, corticospinal, and reticulospinal tracts, an image of these pathways was overlaid on the lesioned hemicord and a spared percentage of either 0%, 25%, 50%, 75%, or 100% was determined for each rat. A similar procedure was completed to determine spared gray matter. The range of lesion sizes was balanced between treatment groups. Variable lesions were included in this study to assess the role of lesion size on functional benefits promoted by optogenetic stimulation.

*Immunohistochemistry*

Every 12th section from the rostral, lesion, and caudal blocks was investigated for GAP-43, laminin, VGlut1, VGlut2, GAD65, and GAD67. Sections were incubated in phosphate-buffered saline (PBS) mixed with 1% normal donkey serum (DS; Abcam, Cambridge, UK) and triton X-100 (T; 1% DS-PBS-T; Sigma-Aldrich) for 3 × 10 min on a shaker table at room temperature. Tissue was then incubated in a blocking solution of 5% DS-PBS-T for 1 h at room temperature. Sections were transferred to an overnight incubation in diluted primary antibody at 4 °C on a shaker table. Specific antibodies and dilutions include: GAP-43 (1:250; Millipore; AB5312), Laminin (1:500; Sigma; L9393), VGLUT1 (1:5000; Millipore; AMAB 91041), VGLUT2 (1:1000; Abcam; AB79157), GAD65 (1:500; Millipore; MAB351), and GAD67 (1:1000; Millipore; MAB5406). The following day, sections were washed with 1% DS-PBS-T for 3 × 10 min. Then, sections were incubated first in a biotinylated secondary antibody (1:200; Jackson ImmunoResearch Labs; 715-065-151) for 1 h on a shaker table at room temperature, followed by streptavidin-680 (1:50; Life Technologies; S32358). Sections were then washed 3 × 10 min with PBS, mounted onto Superfrost plus slides (Fisher Scientific) and coverslipped with PVA mountant (Sigma-Aldrich). Sections were viewed on a Zeiss Axio Zoom microscope (Zeiss; Jena, Germany).

GAP-43 quantitative analysis of axons: The number of GAP-43-labeled fibers was determined using FIJI open-source software (Schindelin *et al* 2012) in 16 spinal sections taken from areas rostral to the lesion site (C2–C3), the lesion site (C4–C5), and caudal to the lesion site (C6–C7), using four sections per spinal area. Each section was imaged using a Zeiss Axio Zoom microscope (Zeiss; Jena, Germany) at 0.51 pixels *μ*m^-1^ using the exposure range indicator to normalize exposure across sections. For each section, the spinal cord was separated into ten regions of interest: (1) ipsilateral dorsal, (2) contralateral dorsal, (3) ipsilateral lateral, (4) contralateral lateral, (5) ipsilateral ventral, (6) contralateral ventral, (7) ipsilateral dorsal gray matter, (8) contralateral dorsal gray matter, (9) ipsilateral ventral gray matter, and (10) contralateral ventral gray matter. Values were averaged across the four sections and then across the respective group.

*Analysis in the white matter* : GAP-43-labeled fibers are easily visualized in the white matter. In these regions, the brightness/contrast was toggled within FIJI (Schindelin *et al* 2012) to accurately highlight GAP-43-labeled fibers within each region of interest. The edges of each fiber were then defined, and the image made binary before using the particle analysis function to count the number of fibers within each region. The particle analysis function allows for the automatic quantification of particles within a given image and region of interest (Grishagin 2015). This function allows for the inclusion/exclusion of particles of specific sizes and shapes for more accuracy. Our analysis included all particles that were >5 pixels, which excluded any non-specific labeling or contamination that may be present on the tissue. We also included all shapes in the analysis (circularity 0–1) as longitudinally traveling fibers appear as circles in our sections and transverse fibers appeared as lines.

*Analysis in the gray matter* : GAP-43-labeled fibers were difficult to identify in the gray matter as we were unable to decipher individual fibers, so a different approach was used for this region. Similar to Jones *et al*, we instead quantified the mean gray area in FIJI, which identifies the level of fluorescence within a given section and region of interest (Jones *et al* 2002). Images were analyzed in grayscale. The mean gray area was determined for each section within the gray matter regions-of-interest in stained sections as well as a control section for each rat and spinal area. The mean gray area from a specific control section was subtracted from the relevant stained section average to account for background fluorescence.

Laminin quantitative analysis for vessel counts: The number of laminin-labeled vessels was determined in a similar way as GAP-43 fiber counts in the white matter. A fixed plane of sectioning was used across all rats. Sixteen sections were analyzed in each rat, with four sections analyzed in the region of the cord rostral to the lesion, four sections containing the lesion site, and four sections caudal to the lesion site where stimulation occurred. Sections were imaged using a Zeiss Axio Zoom microscope (Zeiss; Jena, Germany) using the exposure range indicator to normalize exposure across each section. Images were captured at a magnification of 0.50 pixels *μ*m^−1^. Vessel counts were determined within the same ten regions of interest using FIJI software (Schindelin *et al* 2012). The brightness/contrast was toggled within FIJI to accurately highlight laminin-labeled vessels in each region of interest. Vessel edges were then defined, and images made binary. Particle analysis was then completed to determine the number of vessels within each region.

Quantifying transduced GABAergic and glutamatergic neurons: two sections from the caudal segments of five stimulated rats were imaged using a Zeiss Axio Zoom microscope at a magnification of 0.18 *μ*m/pixel. Sections were analyzed using FIJI (Schindelin *et al* 2012) to determine co-labeling of YFP-labeled synaptic particles (virus) and either VGLUT1/2 or GAD65/67. The number of co-labeled synaptic particles for each type was then divided by the total number of YFP-labeled synaptic particles to determine the percentage of glutamatergic and GABAergic synapses that were virally transduced and likely stimulated via optogenetics.

Experimental design and statistical analysis: Due to the small sample size in the unstimulated (*N* = 5) and optogenetically stimulated (*N* = 6) groups, non-parametric tests were used to assess statistical significance. The Mann–Whitney U-test was used for mean comparisons on the FRT (a total of six tests were performed), IBB (a total of six tests performed), GAP-43 and laminin (30 tests were performed for each antibody). The Wilcoxon rank-sum was used for paired-wise tests to compare GAP-43 and laminin across the rostral, lesion, and caudal regions of the cord within groups (a total of 30 tests were performed for each segment). To identify outliers, the interquartile range was determined for each data set and any data points falling 1.5 × above or below this range were considered outliers (Sunitha *et al* 2014, Vinutha *et al* 2018). All tests were performed using SPSS software version 28.0.0.0 (IBM, Armonk, NY).

## 3. Results

Optogenetic stimulation enhances recovery of the forelimb reaching task

**Figure 2.**
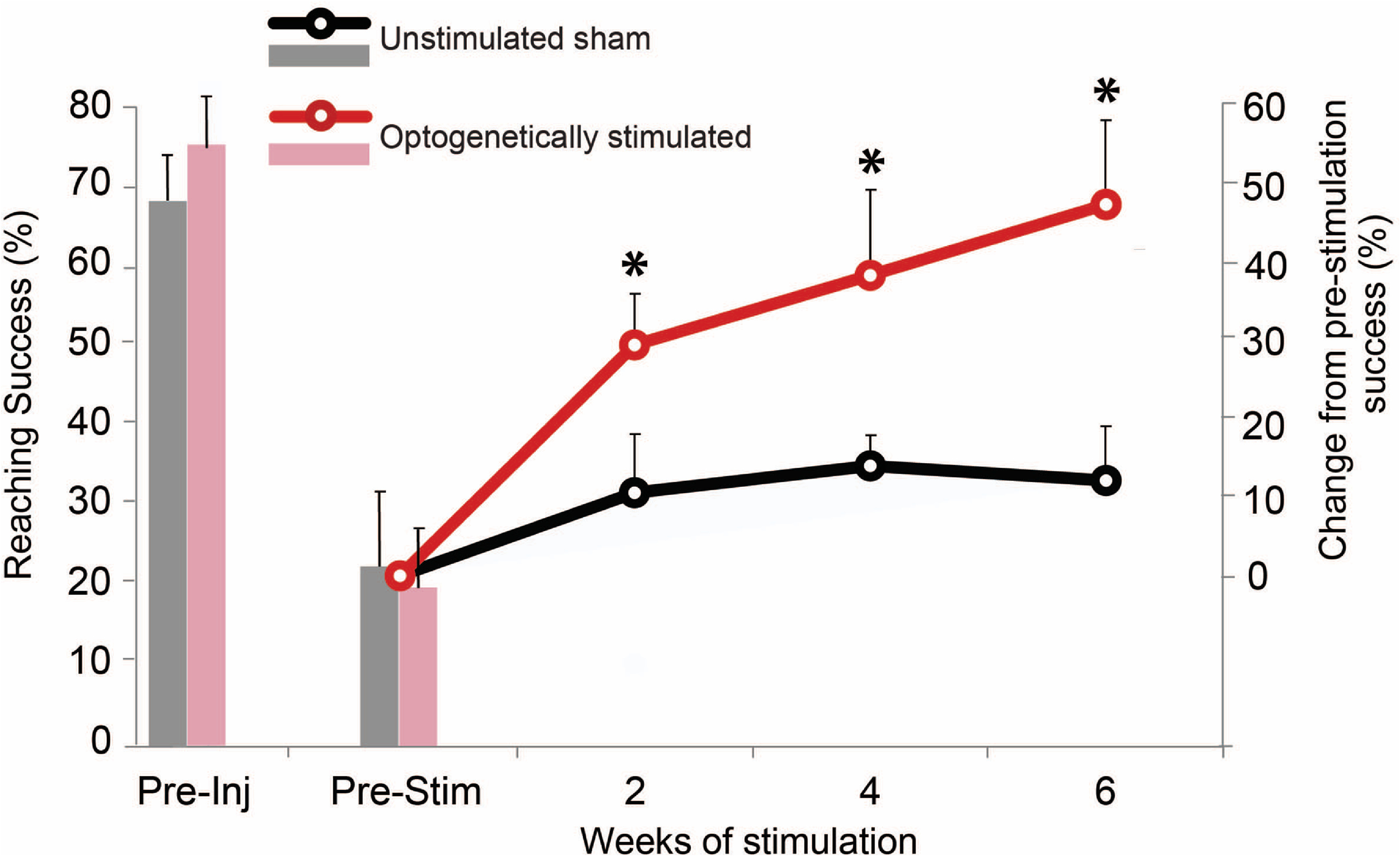
Optogenetic stimulation enhances recovery on the forelimb reaching task. (A) Rats that received optogenetic stimulation demonstrated significantly greater recovery on the FRT compared to unstimulated rats (* = *p* < 0.05). The bar graphs depict the average success before injury and stimulation (five weeks post-injury). The line graph is the average difference from pre-stimulation beginning six weeks post-injury. Optogenetically stimulated animals recover to near pre-injury levels following six weeks of treatment.

Prior to the start of stimulation at six weeks post-injury, the group average of successful reaches on the FRT at five weeks post-injury was 22% ± 0.09% and 20% ± 0.07% for the unstimulated and optogenetically stimulated groups, respectively (figure 2). This indicates similar functionality on this task just before stimulation treatment began (Mann–Whitney U-test; *p* = 0.927).

Within two weeks of stimulation (seven weeks post-injury), animals receiving optogenetic stimulation recovered significantly more forelimb function (29% ± 0.05% above pre-stimulation function) compared to the unstimulated group (4% ± 0.02% from pre-stimulation function; figure 2; Mann–Whitney U-test; *p* = 0.009). Following four weeks of optogenetic stimulation, animals receiving optogenetic stimulation had recovered a total of 38% ± 0.09% above pre-stimulation function, significantly more than the recovery observed in the unstimulated group (14% ± 0.04%, figure 2; Mann–Whitney U-test; *p* = 0.047). The optogenetically stimulated group continued to demonstrate significantly greater success (47% ± 0.09% above pre-stimulation function) compared to the unstimulated group for the final two weeks of stimulation (12% ± 0.04%, figure 2; Mann–Whitney U-test; *p* = 0.014). At this final time point, an outlier was removed from the optogenetic stimulation group as they were 1.5 × below the interquartile range. Inclusion of the outlier adjusts the *p*-value to 0.116 at this final time point only.

Changes to specific limb characteristics during the FRT within each group, such as digit movement or aim, did not suggest any specific aspects of limb movement were responsible for the improved reaching success in the stimulated group (figure S2). Instead, each rat seemed to utilize different movement strategies to successfully retrieve pellets. Similarly, there were no significant group differences on the IBB or LUAT tasks (figures S3–S5). The optogenetic group, however, trended toward an increase in volar contact compared to pre-stimulation at nine weeks post-injury on the IBB that was not seen in the unstimulated group (figure S3; Mann–Whitney U-test; *p* = 0.147). This was determined from IBB video analysis.

Effectiveness of optogenetic stimulation on functional recovery depends on lesion magnitude and spared tissue

**Figure 3.**
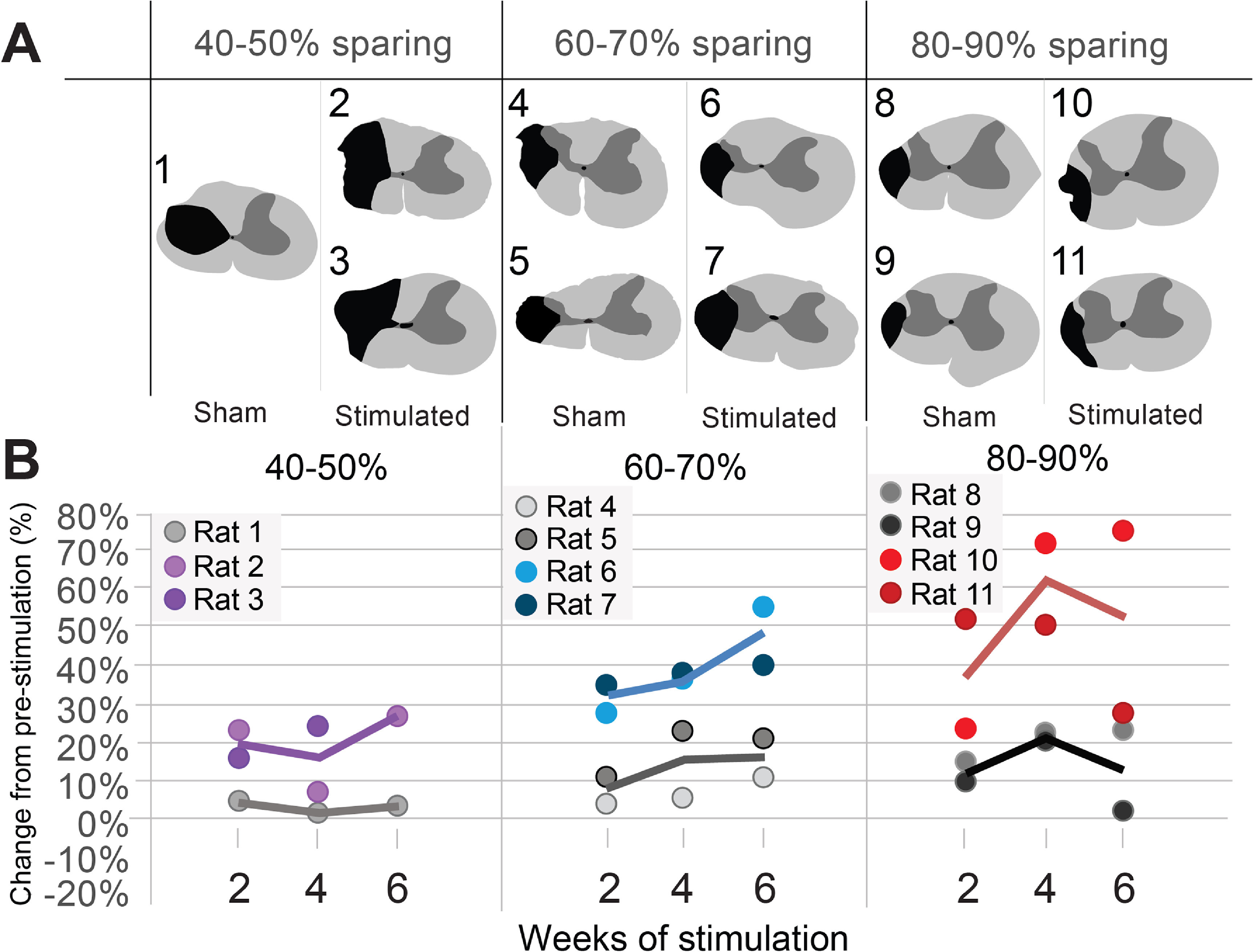
Optogenetic stimulation may be more effective in rats with more spared tissue. (A) Shows the range of injury magnitudes included in each group shown as percent sparing of the injured hemicord. To identify the effect of spared tissue on functional recovery and its interaction with optogenetic stimulation, rats with similar amounts of sparing at the lesion epicenter are grouped together. Renderings of the injury epicenter show spared white matter in light gray, spared gray matter in dark gray, and lesioned tissue in black. (B) Scatter plot graphs depict the difference in FRT success between the sparing groups after 2, 4, and 6 weeks of optogenetic (colored) or sham (grey) stimulation. Each point depicts an individual rat. Line graphs depict the group average for each lesion range (%). Results indicate that optogenetic stimulation is more effective in rats with more sparing following as little as two weeks of stimulation.

Consistent injury magnitudes are difficult to achieve, especially in contusion models. Nonetheless, it is important to understand the effect of injury size on the overall functional outcomes of a given treatment. To do this, we determined the percent sparing of the ipsilesional hemicord, as well as three main motor control pathways: rubrospinal tract, corticospinal tract, and reticulospinal tract. The same analysis was also completed for the gray matter. Rats within each group were secondarily grouped based on overall hemicord sparing (40%–50%, 60%–70%, 80%–90%; figure 3(A)). Performance on the FRT was then compared based on hemicord sparing (figure 3).

Three rats in the 40%–50% spared group had the most severe injuries in the study. Overall, there was no sparing of the rubrospinal tract, modest sparing of the corticospinal tract, complete sparing of the reticulospinal tract and no spared gray matter (figure 3(A)). Over the course of six weeks of stimulation, the two optogenetically stimulated rats had higher individual scores compared to the unstimulated rat. The average success rate for the optogenetically stimulated rats in this most injured group was higher than the unstimulated rat, but not as enhanced as optogenetically stimulated rats with more moderate injuries (figure 3(B)). At six weeks post-injury, one score from the optogenetically stimulated rats in this lesion group fell into outlier range and was excluded from the data.

Four animals comprised the moderate lesion group, defined as 60%–70% tissue sparing. There was no sparing of the rubrospinal tract, complete sparing of the corticospinal and reticulospinal tracts and minor sparing of the gray matter (figure 3(A)). After six weeks of treatment or sham stimulation, the individual scores for the optogenetically stimulated rats were 26%–44% higher than scores for the unstimulated rats (figure 3(B)). The individual scores for the stimulated rats also fell between the scores for the stimulated rats with more or less severe injuries (figure 3(B)). A similar trend was noted for the average success rates (figure 3(B)).

The final four animals comprised the least severe lesion group, defined as 80%–90% tissue sparing. They had no sparing of the rubrospinal tract, complete sparing of the corticospinal and reticulospinal tracts, and nearly complete sparing of the gray matter (figure 3(A)). Overall, the optogenetically stimulated rats had 30%–53% higher individual success scores compared to unstimulated rats throughout the treatment period (figure 3(B)). On the sixth week of treatment, one rat from each group exhibited reduced performance (figure 3(B)). Nonetheless, the average success rate in the optogenetically stimulated rats for the least severe lesion group was higher than the unstimulated group with similarly sized lesions, and highest compared to the other stimulated rats.

Overall, these findings indicate that optogenetic stimulation using the current parameters is likely more effective on smaller lesions with more spared tissue. Notably, the greatest difference in tissue sparing across the three lesion magnitude groups is within the gray matter, suggesting this region may play a key role in supporting optogenetic-induced functional recovery. Additional rats for each lesion magnitude category must be studied in the future to determine the true impact of lesion magnitude on treatment effect.

Optogenetic stimulation enhances cervical axonal growth

**Figure 4.**
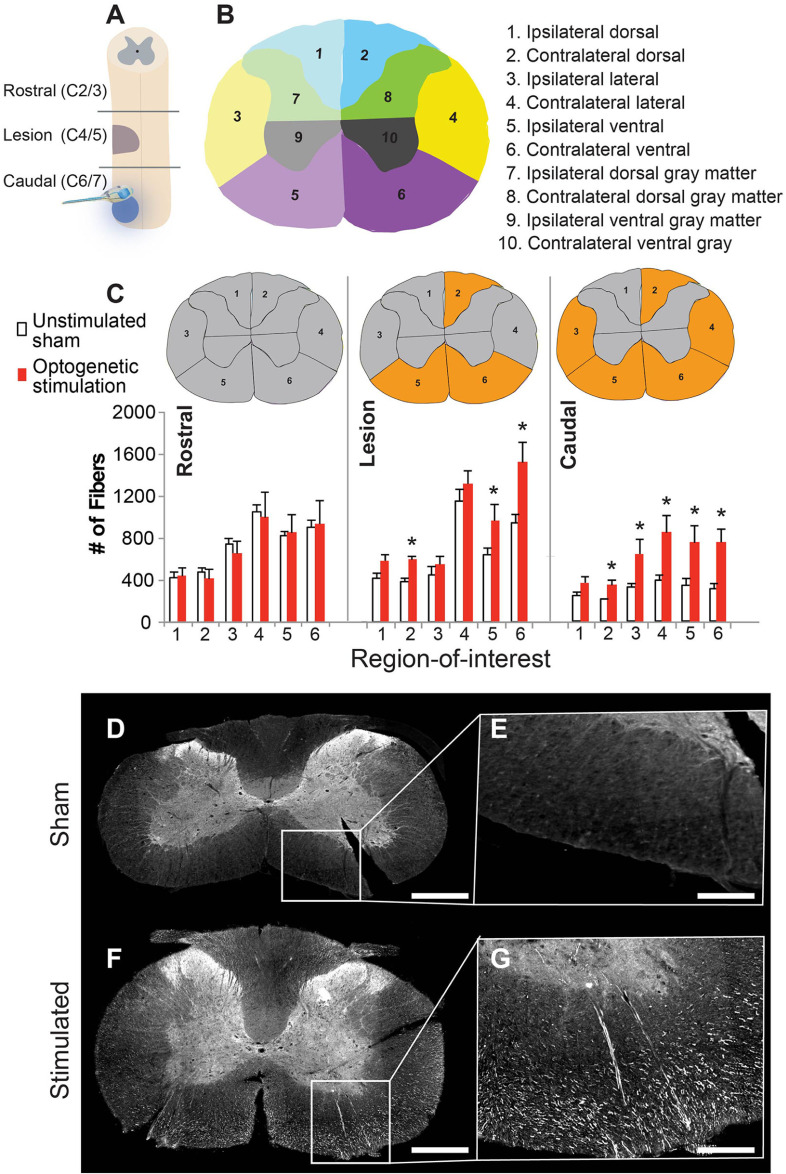
Optogenetic stimulation enhances axonal growth within the injured spinal cord. (A) Schematic showing location of the spinal injury (purple), viral injections (blue dot), and implant placement/optogenetic stimulation (same site as viral injection) relative to the ‘rostral’, ‘lesion’, and ‘caudal’ spinal cord regions of analysis. (B) Immunoreactivity against GAP-43 was used to identify new axonal growth in ten regions of interest, depicted in panel (B). (C) Bar graph depicting the # of GAP-43-labeled fibers in each segment and region-of-interest, (gray matter regions not depicted—see methods section for explanation) (* = *p* < 0.05). Spinal cord cross sections depict location of either no change (gray) or a significant change in GAP-43 (orange) across the two groups. Representative images of GAP-43 labeling (white) taken from the caudal spinal cord from an unstimulated animal (D), (E) and optogenetically stimulated rat (F), (G). The high magnification panels (E), (G) were taken from the contralateral side. Scale bar is 1 mm for D, F and 300 *μ*m for E, G.

Changes in axonal growth were determined using a GAP-43 antibody, a marker of new axonal growth. GAP-43 was quantified across three general cervical regions: spinal segments rostral to the lesion site (C2/3), at the level of the lesion (C4/5), and caudal to the lesion (C6/7) at the site of stimulation (figure 4(A)). Each spinal cord cross-section was divided into ten regions of interest (see Methods and figure 4(B)).

There was a significant increase in GAP-43-labeled axons in the lesion spinal cord segments of optogenetically stimulated rats compared to the unstimulated animals within three regions-of-interest denoted by the asterisks in figure 4(C) (table 1(A)). The caudal spinal cord segments of the stimulated group also contained a significant increase in GAP-43-labeled axons within five regions-of-interest (figures 4(C)–(G); table 1(A)). No significant differences in GAP-43-labeled axons between groups were detected in the rostral spinal cord segments (figure 4(C)) or in the cervical gray matter (figure S1).

**Table 1.** GAP-43 is significantly enhanced following optogenetic stimulation. The Mann–Whitney U-test was used to determine significant differences between groups within each region-of-interest (A). The Wilcoxon signed-rank test was used to determine significant differences within groups across different spinal segments (B). Green-shaded boxes highlight a statistically significant increase, while red shaded boxes highlight a statistically significant decrease (*p* < 0.05).

A		
UNSTIMULATED SHAM VS. OPTOGENETICALLY STIMULATED		
Rostral	Ipsilateral	Contralateral
Dorsal white matter	0.806	0.624
Lateral white matter	0.806	0.624
Ventral white matter	1.000	1.000
Dorsal gray matter	0.624	0.624
Ventral gray matter	0.327	0.624
Lesion	Ipsilateral	Contralateral
Dorsal white matter	0.076	0.009
Lateral white matter	0.175	0.076
Ventral white matter	0.016	0.009
Dorsal gray matter	0.347	0.116
Ventral gray matter	0.465	0.076
Caudal	Ipsilateral	Contralateral
Dorsal white matter	0.076	0.047
Lateral white matter	0.047	0.028
Ventral white matter	0.028	0.009
Dorsal gray matter	0.463	0.463
Ventral gray matter	0.465	0.530

B					
UNSTIMULATED SHAM			OPTOGENETICALLY STIMULATED		
Rostral versus Lesion	Ipsilateral	Contralateral	Rostral versus Lesion	Ipsilateral	Contralateral
Dorsal white matter	0.465	0.144	Dorsal white matter	0.043	0.080
Lateral white matter	0.068	0.465	Lateral white matter	0.080	0.138
Ventral white matter	0.144	0.068	Ventral white matter	0.225	0.043
Dorsal gray matter	0.144	0.144	Dorsal gray matter	0.080	0.043
Ventral gray matter	0.465	0.273	Ventral gray matter	0.080	0.043
Rostral versus Caudal	Ipsilateral	Contralateral	Rostral versus Caudal	Ipsilateral	Contralateral
Dorsal white matter	0.068	0.068	Dorsal white matter	0.500	0.500
Lateral white matter	0.068	0.068	Lateral white matter	0.500	0.500
Ventral white matter	0.068	0.068	Ventral white matter	0.345	0.225
Dorsal gray matter	0.465	0.273	Dorsal gray matter	0.686	0.893
Ventral gray matter	0.465	0.465	Ventral gray matter	0.686	0.686
Lesion versus Caudal	Ipsilateral	Contralateral	Lesion versus Caudal	Ipsilateral	Contralateral
Dorsal white matter	0.043	0.043	Dorsal white matter	0.043	0.043
Lateral white matter	0.345	0.043	Lateral white matter	0.893	0.043
Ventral white matter	0.043	0.043	Ventral white matter	0.043	0.043
Dorsal gray matter	0.893	0.225	Dorsal gray matter	0.500	0.225
Ventral gray matter	0.585	0.138	Ventral gray matter	0.500	0.345

We next compared the number of GAP-43-labeled fibers among the three different spinal segments within each group: (1) rostral to the lesion, (2) centered on the lesion, and (3) caudal to the lesion. In the unstimulated group, the number of GAP-43-labeled fibers was similar between the rostral versus lesion segments, and rostral versus caudal segments. However, a significant decrease in GAP-43 fibers was noted when comparing lesion segments to caudal segments (figure 4(C); table 1(B)).

By contrast, optogenetically stimulated rats had significantly more GAP-43-labeled fibers in the lesioned segment compared to the rostral segment (figure 4(C); table 1(B)). While there was a substantial decrease in GAP-43 fibers in the caudal segments of the unstimulated group, there were no differences between the rostral and caudal segments in the optogenetically stimulated group (figure 4(C); table 1(B)). This indicates that optogenetic stimulation likely promoted new growth within the caudal segments that did not occur in the unstimulated group.

Optogenetic stimulation upregulates new vascularization throughout the cervical spinal cord

**Figure 5.**
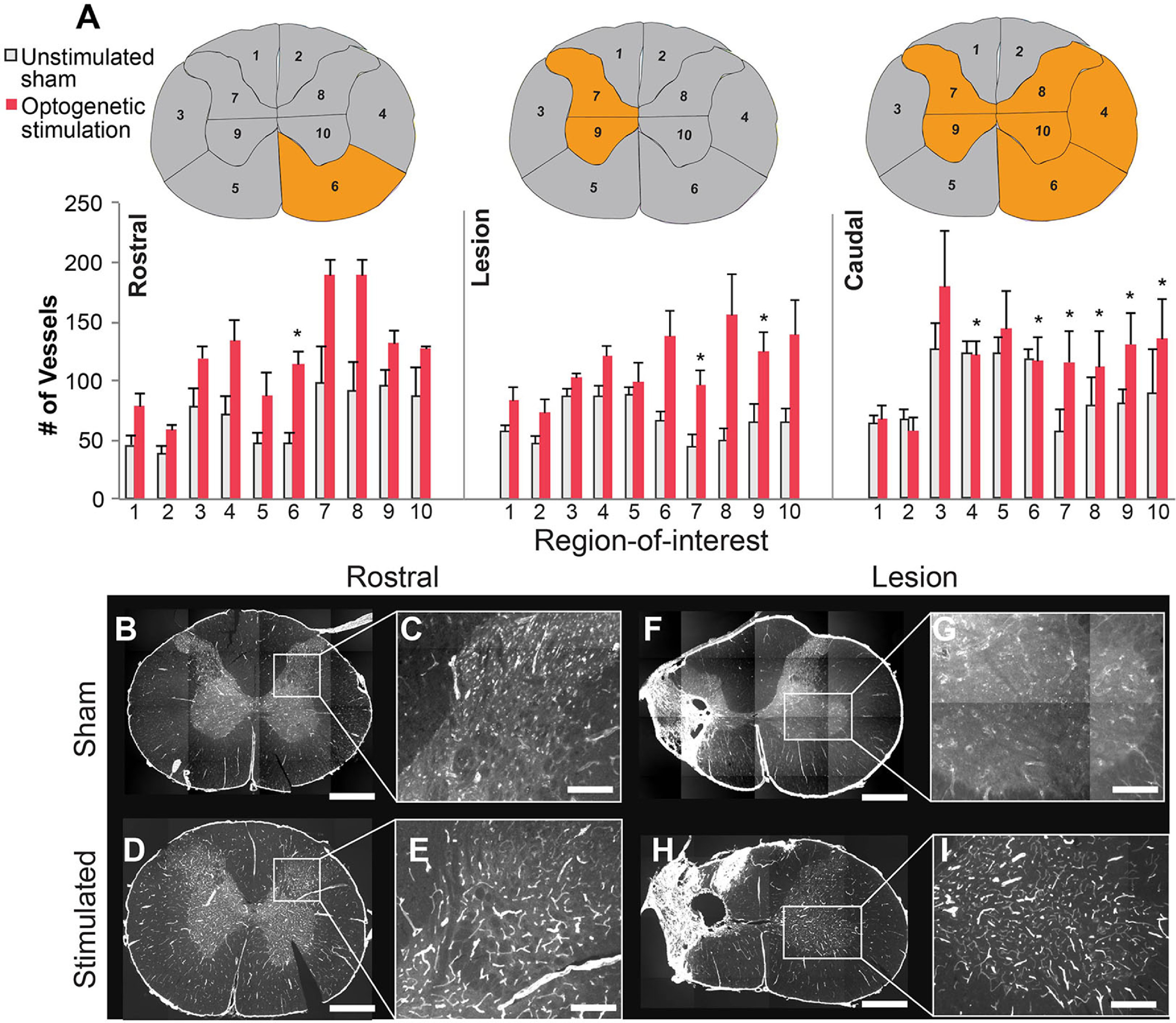
Optogenetic stimulation promotes increased vascularization in the injured spinal cord. (A) Vasculature was quantified in similar regions of interest as GAP-43 within the rostral, lesion, and caudal spinal cord segments using laminin immunoreactivity. (B) Bar graphs depict the number of vessels containing laminin in the rostral, lesion, and caudal segments for the unstimulated and stimulated groups; (* = *p* < 0.05). Spinal cord cross-sections depict location of either no change in laminin (gray) or a significant change (orange) across the two groups. Representative images taken from the rostral segments (B), (C) and lesion segments with anti-laminin (white) (F), (G) from unstimulated rats, as well as rostral and lesion segments from the optogenetically stimulated rats, respectively (D), (E), (H), (I). The higher magnification panels (C), (E), (G), (I) were taken from the contralateral side. Scale bars for B, D, F, H are 1 mm, 350 *μ*m for C, E, and 200 *μ*m for G, I.

Vasculature was quantified in the same regions of interest as GAP-43 within the segments rostral to the lesion, centered on the lesion, and caudal to the lesion using laminin immunoreactivity. The pattern of increased laminin was similar to that observed for GAP-43. In the rostral spinal segment, there was one region-of-interest that contained significantly more vasculature in the animals treated with optogenetic stimulation (figures 5(A)–(E); table 2(A)).

**Table 2.** Laminin is significantly enhanced following optogenetic stimulation. The Mann–Whitney U-test was used to determine significant differences between groups within each region-of-interest (A). The Wilcoxon signed-rank test was used to determine significant differences within groups across different spinal segments (B). Green-shaded boxes highlight a statistically significant increase, while red shaded boxes highlight a statistically significant decrease (*p* < 0.05).

A		
UNSTIMULATED SHAM VS. OPTOGENETICALLY STIMULATED		
Rostral	Ipsilateral	Contralateral
Dorsal white matter	0.05	0.142
Lateral white matter	0.086	0.05
Ventral white matter	0.142	0.014
Dorsal gray matter	0.086	0.05
Ventral gray matter	0.086	0.221
Lesion	Ipsilateral	Contralateral
Dorsal white matter	0.465	0.402
Lateral white matter	0.117	0.465
Ventral white matter	0.117	0.602
Dorsal gray matter	0.016	0.175
Ventral gray matter	0.009	0.076
Caudal	Ipsilateral	Contralateral
Dorsal white matter	0.117	0.117
Lateral white matter	0.175	0.028
Ventral white matter	0.602	0.009
Dorsal gray matter	0.028	0.016
Ventral gray matter	0.028	0.028

B					
UNSTIMULATED SHAM			OPTOGENETICALLY STIMULATED		
Rostral versus Lesion	Ipsilateral	Contralateral	Rostral versus Lesion	Ipsilateral	Contralateral
Dorsal white matter	0.068	0.465	Dorsal white matter	0.893	0.500
Lateral white matter	0.715	0.465	Lateral white matter	0.225	0.345
Ventral white matter	0.068	0.273	Ventral white matter	0.500	0.225
Dorsal gray matter	0.144	0.068	Dorsal gray matter	0.043	0.225
Ventral gray matter	0.273	0.068	Ventral gray matter	0.686	0.893
Rostral versus Caudal	Ipsilateral	Contralateral	Rostral versus Caudal	Ipsilateral	Contralateral
Dorsal white matter	0.144	0.144	Dorsal white matter	0.893	0.893
Lateral white matter	0.144	0.144	Lateral white matter	0.080	0.893
Ventral white matter	0.068	0.068	Ventral white matter	0.138	0.345
Dorsal gray matter	0.144	1.000	Dorsal gray matter	0.043	0.500
Ventral gray matter	0.465	0.068	Ventral gray matter	0.686	0.345
Lesion versus Caudal	Ipsilateral	Contralateral	Lesion versus Caudal	Ipsilateral	Contralateral
Dorsal white matter	0.500	0.225	Dorsal white matter	0.078	0.225
Lateral white matter	0.138	0.080	Lateral white matter	0.080	0.345
Ventral white matter	0.043	0.043	Ventral white matter	0.138	0.893
Dorsal gray matter	0.686	0.138	Dorsal gray matter	0.225	0.138
Ventral gray matter	0.080	0.500	Ventral gray matter	0.686	0.225

In the lesion spinal segment, there were two regions-of-interest containing significantly more vasculature in the optogenetically stimulated rats (figure 5(A); table 2(A)). Within the caudal spinal segment where stimulation occurred, six regions-of-interest showed significantly more vasculature compared to unstimulated rats (figures 5(A), (F)–(I); table 2(A)). Comparisons between the different segments within each group did not indicate many segmental differences (figure 5(A); table 2(B)). Overall, these findings indicate that optogenetic stimulation promoted significant new vascularization throughout the cervical spinal cord.

Transduction of neuronal cell-types

**Figure 6.**
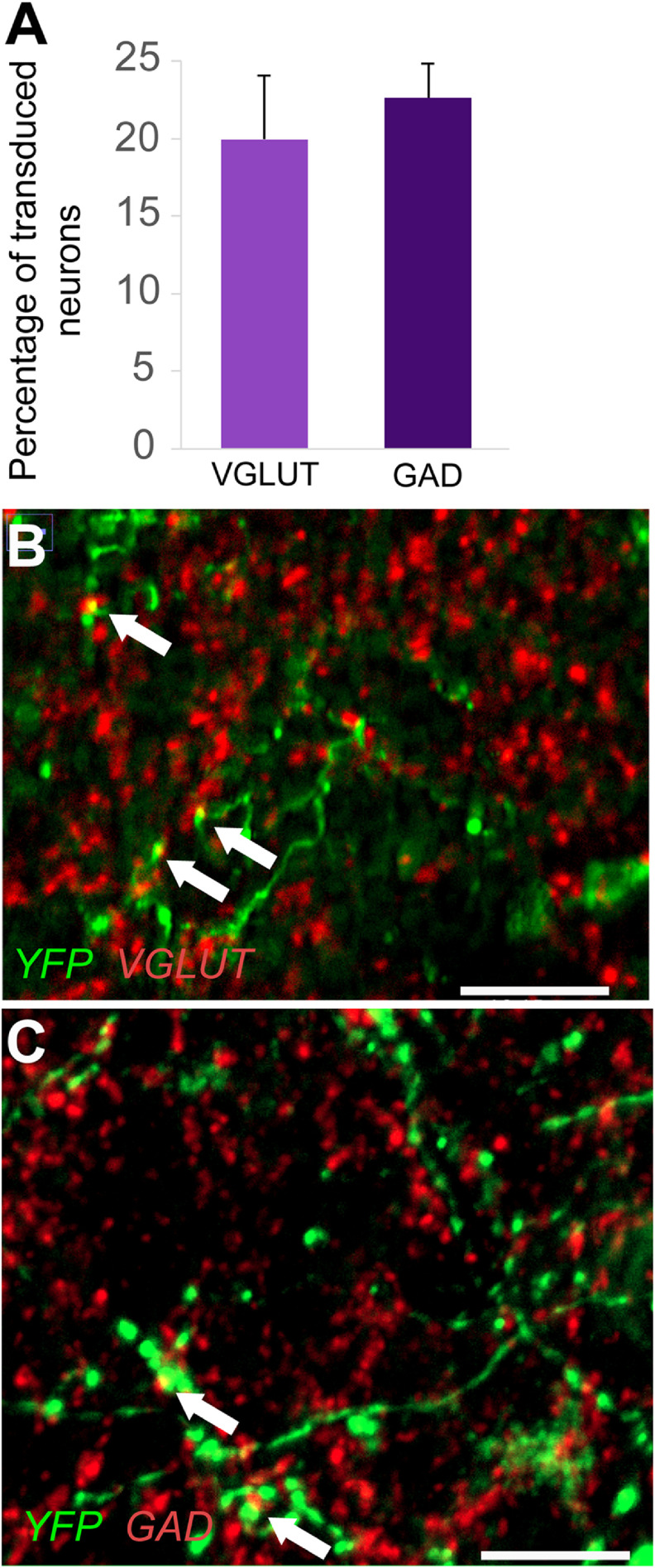
*AAV2-hSyn-ChR2-YFP* viral vector transduces both glutamatergic and gabaergic synapses. (A) Bar graph depicts the percentage of co-labeled transduced synapses (*AAV2-hSyn-ChR2-YFP*) with VGLUT1/2 and GAD65/67 immunoreactivity. (B) A representative image of VGLUT (red) and YFP (green) labeling, as well as of (C) GAD (red) and YFP (green) labeling are also shown. Scale bars are 18 *μ*m.

We investigated the proportion of glutamatergic and GABAergic synapses transduced by the optogenetic virus and thus likely stimulated with light during optogenetic stimulation. We saw 20% ± 0.04% glutamatergic and 22% ± 0.02% GABAergic transduced synapses (figure 6). These values describe the general balance of glutamatergic versus GABAergic synapses, but do not specify the overall number of these respective cell types. This suggests a balanced combination of excitatory and inhibitory optogenetic activation occurred within the stimulated rats.

Location of viral transduction in the spinal cord

**Figure 7.**
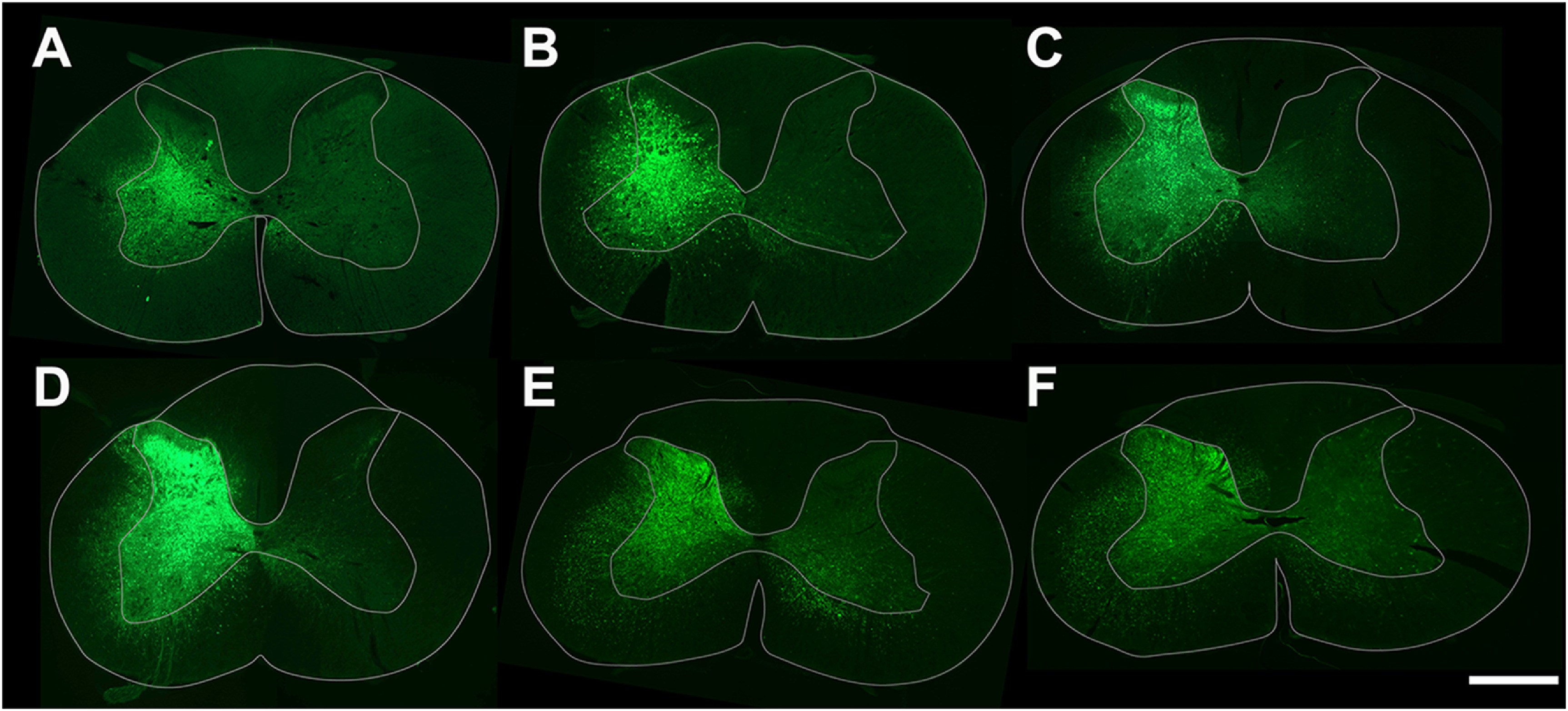
Viral transduction patterns in stimulated rats suggests stimulation occurred in similar laminae. (A)–(F) Images taken of the section containing the most transduction for each of the optogenetically stimulated rats. Green fluorescence depicts transduction of *AAV2-hSyn-ChR2-YFP* optogenetic virus. Scale bar is 1 mm.

Each of the rats that received optogenetic stimulation were assessed for overall viral vector transduction (*AAV2-hSyn-ChR2-YFP*) at spinal segment C6. Most of the stimulated rats had viral transduction throughout the hemicord gray matter (figures 7(A)–(F)), with two of the rats having minimal to no transduction in lamina I–III (figures 7(A) and (B)).

## 4. Discussion

This is the first study to describe the functional effects of optogenetic stimulation after cervical SCI. Our overall findings suggest optogenetic stimulation promotes robust functional recovery that is affected by lesion magnitude and supported in part by new axonal growth and angiogenesis. Several key aspects of these findings are discussed below.

Effects of optogenetic stimulation on forelimb function

The current study shows significantly enhanced recovery of forelimb reaching in rats that received optogenetic stimulation. While this is the first study to investigate the therapeutic effects of long-term optogenetic stimulation in the cervical spinal cord, a parallel study looked at the effects of *bioluminescent* optogenetic stimulation after SCI in the lumbar cord (Petersen *et al* 2022). This recent study investigated the effects of two weeks of bioluminescent-optogenetic stimulation of the rat lumbar spinal cord after severe thoracic contusion and reported enhanced BBB scores. Another study investigated the effects of short-term, acute optogenetic stimulation of the rat spinal cord after a C2 hemisection and reported renewed ipsilateral diaphragm activity lasting for ∼24 h (Alilain *et al* 2008). It was also determined that acute, short-term optogenetic stimulation of spinal bladder neurons after SCI produced a unique return of bladder function (Awad *et al* 2013). The success of these studies highlights the robust neuromodulatory capacity of optogenetic stimulation and suggests its therapeutic potential following SCI.

Stimulation effects on unpaired tasks

The present study found significantly enhanced recovery on the forelimb reaching task, but not the IBB and LUAT tests. This may be due to pairing stimulation with only the FRT, leading to the formation of new circuitry tailored to FRT performance via Hebbian plasticity in which ‘neurons that fire together wire together’ (Hebb 1949). Specifically, stimulation occurring during the attempted performance of a functional task likely enhances the formation of new circuitry supportive of that task. Even if the animal struggles to perform the task initially, some spared circuitry could still be activated and promote new functionally-relevant circuits. Prior work demonstrated that spinally injured rats showed significantly better recovery on the FRT after receiving synchronized intraspinal stimulation triggered by activity of forelimb muscles (McPherson *et al* 2015). Our findings suggest that this effect may also occur through pairing performance of a task with optogenetic stimulation. A recent study found that pairing optogenetic stimulation of the brain with rehabilitation produced greater recovery post-stroke than rehabilitation or stimulation alone (Conti *et al* 2022). Interestingly, although we found significantly more reaching success on the FRT in stimulated rats, we did not observe any significant differences in specific limb features associated with the reaching task between the stimulated and unstimulated animals. This suggests that each stimulated rat utilized a different limb strategy for successfully reaching and grasping the pellets, which averaged out changes in specific limb features across animals. Notably, while we did not see recovery on the IBB, we did see a trend toward recovery on one of the subcategories that shared similarities with movement functionality to the FRT: volar contact support. This further suggests that new circuitry in the current study may have been shaped to support reaching and grasping-related behaviors.

Location of induced axonal growth

**Figure 8.**
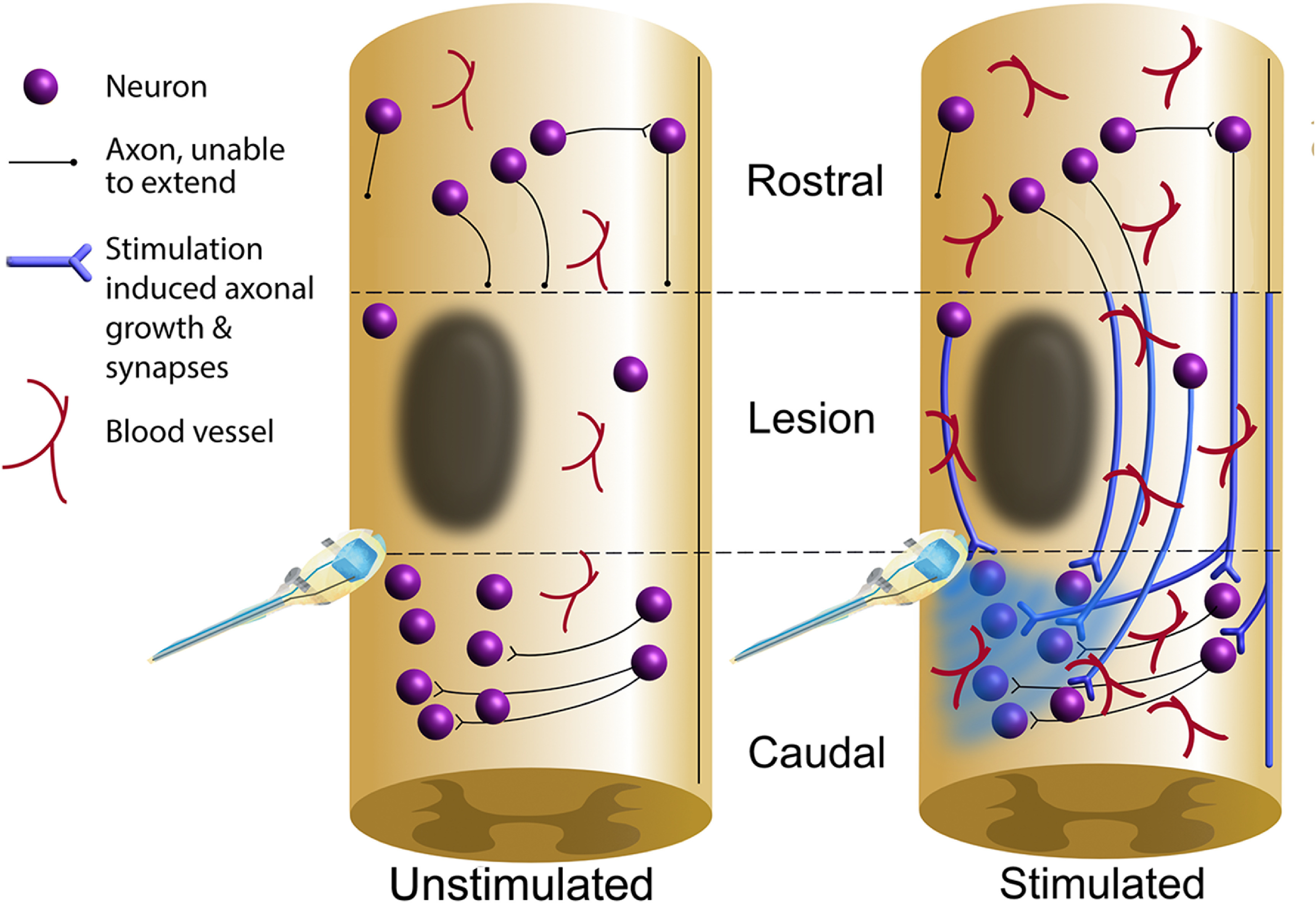
Optogenetic stimulation may promote the formation of new lesion-bridging circuitry. Schematic diagram depicting hypothesized axonal plasticity and angiogenesis occurring in the cervical spinal cord of the unstimulated animals compared to the optogenetically stimulated animals based on findings from the current study. Results suggest that while both unstimulated and stimulated rats contain *similar levels of axonal growth rostral to the lesion*, stimulation promotes significantly more axonal growth in the white matter within the lesioned segments and caudal regions. This may produce a bridge around the lesion to support enhanced functional recovery. Our findings also suggest stimulation enhances angiogenesis throughout the cervical cord.

Optogenetic stimulation increases GAP-43 expression, similar to other long-term optogenetic studies following stroke (Cheng *et al* 2014, Shah *et al* 2017). Notably, the location of this new growth is particularly informative. Our results indicate enhanced growth both within the lesioned and stimulated segments in the optogenetically-stimulated rats (figure 8). This suggests that while both unstimulated and stimulated rats have similar levels of axonal growth above the lesion site, this new growth stops short above the lesioned segments in unstimulated animals. However, in the presence of stimulation this new growth is able to continue traveling caudally around the lesion in the stimulated rats to potentially form a bridge with downstream neurons. Local sprouting within the lesion and caudal regions may also be occurring, synapsing with newly formed circuitry and stimulated neurons. Significantly more axonal growth was noted in the stimulated rats in both the ipsilateral and contralateral hemicords, suggesting that spared pathways likely contribute to the newly formed circuitry. This is in line with previous studies that have reported spared circuitry after SCI to be involved in new circuits and may contribute to the partial restoration of function (Courtine *et al* 2008, Carmel and Martin 2014, García-Alías *et al* 2015, Fink and Cafferty 2016, Jiang *et al* 2016, Hutson and Di Giovanni 2019, Kazim *et al* 2021).

Both unstimulated and stimulated rats share a similar amount of axonal growth within the gray matter, where-as stimulated rats had significantly more GAP-43 in the white matter. This finding highlights the importance of plasticity within the white matter for promoting functional recovery. Propriospinal pathways have been shown in several studies to be particularly plastic after SCI and likely represent a significant portion of new growth induced by optogenetic stimulation (Bareyre *et al* 2004, Courtine *et al* 2008, Flynn *et al* 2011, Côté *et al* 2012, Doperalski *et al* 2020).

The neuronal activation induced by optogenetic stimulation is likely the driving force behind the enhanced axonal growth reported here. Neuronal activation produces numerous neurophysiological effects that ultimately support increased axonal growth (Mondello *et al* 2014). These include upregulating the production of brain-derived neurotrophic factor (BDNF; Al-Majed *et al* 2000), neuroprotection from cyclic adenosine monophosphate-dependent mechanisms of cell death (Shen *et al* 1999, Goldberg *et al* 2002) and upregulation of the activity-regulated inhibitors of death genes (Zhang *et al* 2007, Tan *et al* 2012). Further, optogenetic stimulation *in-vitro* enhances neurite outgrowth in dorsal root ganglia cells (Park *et al* 2015) and axon elongation in motoneurons (Hyung *et al* 2019). An increase in myelin basic protein expressing-Schwann cells was also reported, which led to the initiation of myelination (Hyung *et al* 2019). Additionally, during early nervous system development, neuronal activity guides developing axons towards functionally appropriate targets (Penn and Shatz 1999, Hohnke and Sur 2001, Pan and Monje 2020). Taken together, optogenetic stimulation below the lesion site may both increase axonal growth and help direct that new growth towards nearby functional targets.

Effect of stimulation on vasculature

Studies have shown that neuro-vasculature and endogenous neural activity are tightly coupled such that excitatory neural activity increases blood flow (Cox *et al* 1993, Harder *et al* 1998, Leybaert 2005). Our results indicate increased vasculature throughout the cervical cord in stimulated animals (figure 8). These results are noteworthy as few studies have investigated the effects of exogenous stimulation on angiogenesis within the cord. Interestingly, similar results have been reported in skeletal muscle following electrical stimulation (Adair *et al* 1995, Linderman *et al* 2000, Amaral *et al* 2001, Kanno *et al* 2014, Beugels *et al* 2019).

In addition to the well-known benefits of increased neuro-vasculature, such as supplying tissue with oxygen and nutrients, a tight relationship between neuro-vasculature and improved functional outcomes has been reported (Glaser *et al* 2006, Kaneko *et al* 2006, Ohab *et al* 2006, Yoshihara *et al* 2007). In fact, neuro-vasculature has been shown to act as a scaffold that provides guidance for new axonal growth (Bearden and Segal 2004, Figley *et al* 2013). Interestingly, microvessels within the spinal gray matter are important for reducing cell death and promoting tissue health after injury (Peters *et al* 1993, Raab and Plate 2007). Lastly, increased angiogenesis has been associated with increased metabolic activity (Black *et al* 1987, 1991, Isaacs *et al* 1992, Adair and Montani 2010), indicating a possible increase in metabolic support throughout the cervical spinal cord. Here, we found significantly enhanced vasculature within the ipsilateral gray matter of the lesioned segment in the animals receiving stimulation, as well as within the entire gray matter region near the stimulation site (summarized in figure 8).

It is important to note that since we did not measure vascular volume, our results may reveal only part of the neuro-vasculature changes. Nonetheless, these findings suggest that functional improvements seen after optogenetic stimulation may be partly attributed to increased gray matter vasculature, which could translate into healthier neuronal populations that are more supportive of new circuitry.

Neuron-specific stimulation is a powerful neuromodulator within the spinal cord

Our findings suggest that stimulation of neurons in the absence of direct glial stimulation can promote significant functional recovery after SCI. This is notable as the majority of past therapeutic stimulation studies utilized electrical stimulation, which activates both neurons and glial cells (Roitbak and Fanardjian 1981, Gellner *et al* 2016, Vallejo *et al* 2019, Tsui *et al* 2022). Glial cells, particularly astrocytes which are the largest population of cells in the nervous system (Miller 2018), are involved in promoting neural plasticity by coordinating the formation of synapses and maintaining and strengthening the resulting circuitry (Pfrieger and Barres 1997, Ullian *et al* 2001, Perez-Catalan *et al* 2021). Astrocytes are also involved in synaptic pruning to ensure precise circuitry (Chung *et al* 2013, Neniskyte and Gross 2017, Perez-Catalan *et al* 2021) and shaping neuronal signaling (Perez-Catalan *et al* 2021) through endogenous Ca^2+^ signaling (Cornell-Bell *et al* 1990, Dani *et al* 1992, Newman and Zahs 1997, Lines *et al* 2020, Perez-Catalan *et al* 2021). Microglia also participate in some synaptic pruning (Paolicelli *et al* 2011, Schafer *et al* 2012, Chung *et al* 2013, Hong *et al* 2016). Interestingly, the enhanced axonal growth and functional recovery reported in our current study suggests that direct activation of neurons alone may be sufficient for producing significant functional benefits after spinal injury. It is possible, however, that optogenetic stimulation of neurons may also indirectly activate glial cells. Future studies should explore the isolated activation of glial cells in the absence of neuron stimulation to tease out the importance of each cell type in promoting recovery after SCI.

## 5. Conclusion

The current study demonstrates that optogenetic stimulation has robust neuromodulatory effects after injury, and shows therapeutic promise for treating SCI. Similar findings have been reported in animal models for other neurological diseases including stroke (Shah *et al* 2017, Lu *et al* 2019, Pendharkar *et al* 2021, Conti *et al* 2022), Parkinson’s disease (Magno *et al* 2019, Spix *et al* 2021), depression (Hare *et al* 2019), anxiety (Liu *et al* 2020), and epilepsy (Chiang *et al* 2014). Future studies should begin investigating the roles of individual cell types, populations, and pathways in order to identify the optimal stimulation strategy for restoring function after SCI. Once these mechanisms are understood, optogenetic stimulation could be a promising future therapeutic for SCI.

## Data Availability

The data that support the findings of this study are openly available at the following URL/DOI: https://odc-sci.org.
